# Predictors of Treatment Resistance Across Different Clinical Subtypes of Depression: Comparison of Unipolar vs. Bipolar Cases

**DOI:** 10.3389/fpsyt.2020.00438

**Published:** 2020-05-15

**Authors:** Michele Fornaro, Andrea Fusco, Stefano Novello, Pierluigi Mosca, Annalisa Anastasia, Antonella De Blasio, Felice Iasevoli, Andrea de Bartolomeis

**Affiliations:** ^1^Laboratory of Molecular and Translational Psychiatry, Unit of Treatment-Resistant Psychosis, Section of Psychiatry, University of Naples Federico II, Naples, Italy; ^2^Polyedra Research Group, Teramo, Italy

**Keywords:** treatment-resistance, depression, bipolar disorder, mixed states, mixed features

## Abstract

**Objective:**

Treatment-resistant depression (TRD) and treatment-resistant bipolar depression (TRBD) poses a significant clinical and societal burden, relying on different operational definitions and treatment approaches. The detection of clinical predictors of resistance is elusive, soliciting clinical subtyping of the depressive episodes, which represents the goal of the present study.

**Methods:**

A hundred and thirty-one depressed outpatients underwent psychopathological evaluation using major rating tools, including the Hamilton Rating Scale for Depression, which served for subsequent principal component analysis, followed-up by cluster analysis, with the ultimate goal to fetch different clinical subtypes of depression.

**Results:**

The cluster analysis identified two clinically interpretable, yet distinctive, groups among 53 bipolar (resistant cases = 15, or 28.3%) and 78 unipolar (resistant cases = 20, or 25.6%) patients. Among the MDD patients, cluster “1” included the following components: “Psychic symptoms, depressed mood, suicide, guilty, insomnia” and “genitourinary, gastrointestinal, weight loss, insight”. Altogether, with broadly defined “mixed features,” this latter cluster correctly predicted treatment outcome in 80.8% cases of MDD. The same “broadly-defined” mixed features of depression (namely, the standard Diagnostic and Statistical Manual for Mental Disorders, Fifth Edition—DSM-5—specifier plus increased energy, psychomotor activity, irritability) correctly classified 71.7% of BD cases, either as TRBD or not.

**Limitations:**

Small sample size and high rate of comorbidity.

**Conclusions:**

Although relying on different operational criteria and treatment history, TRD and TRBD seem to be consistently predicted by broadly defined mixed features among different clinical subtypes of depression, either unipolar or bipolar cases. If replicated by upcoming studies to encompass also biological and neuropsychological measures, the present study may aid in precision medicine and informed pharmacotherapy.

## Introduction

Almost half of the patients suffering from a major depressive episode (MDE) fail to achieve a response, despite sequential combination or augmentation treatment strategies, irrespective of the operational definition adopted for treatment-resistant depression (TRD) (1, 2). The rates of treatment-resistant bipolar depression (TRBD) within the course of bipolar disorder (BD) can even exceed those documented for major depressive disorder (MDD) and present a differential pattern of response to different medications, indeed (3). Both TRD (4) and TRBD (5, 6) pose a significant clinical and societal burden, soliciting the assessment of putative predictors using clinical, neuropsychological, neuroimaging, and genetic measures (7, 8). However, the general failure to find sensitive and specific predictors of treatment response in MDD and BD, either current- of future-state markers (9), is in part, due to the heterogeneity of these conditions, which range from biologically determined to event-dependent conditions (10). The initial hype about digital phenotyping has been tempered by increasing awareness of the significant technical and analytical difficulties hindering translational change (11, 12) most likely due to differential neurobiology underpinning MDD (13, 14) and BD (15–17). To date, no single biological marker stood-out in selecting the best initial antidepressant treatment for a given individual, despite promising evidence coming from recent pharmacogenomics studies (18). Clinical risk stratification models for TRD, incorporating baseline sociodemographic and clinical features (19), preceded algorithm-based approaches proposed for TRBD (20). For MDD, a cluster of variables including marital status, insomnia, psychosocial impact, trauma, education, energy, disorder recurrence, comorbidity, race, and severity of current MDE resulted in a positive predictive value of TRD =.61 and a negative predictive value =.69, consistently across males and females and primary- vs. specialty care settings (19). Over the past decade, findings from the Group for the Study of Resistant Depression (GSRD), a multinational European research consortium, pointed toward strong effects of clinical variables on treatment outcome of MDD (21), as well as specific genetic signature (22). This is particularly relevant considering that, when considered separately, the potential predictors of TRD usually showed odd ratios (ORs) around 1.5, and thus, were not applicable as univocal hints in the clinical setting (23, 24). Nonetheless, four potent clinical predictors of TRD emerged in a validation study that showed a collective predictive accuracy of 87%. These latter include symptom severity, suicidal risk, comorbid anxiety disorder, and the lifetime number of MDE episodes (25–27). Overall, symptom severity, suicidal risk, higher number of lifetime of MDEs, and comorbid anxiety disorder were consistently replicated as the most prominent risk factors for different stages of TRD in large-sampled studies (28).

Machine learning approaches may, however, further increase the detection of TRD (29, 30), as reinforced by the 2019 report by the GSRD study group for TRD (22).

Concerning BD, clinical predictors of “full clinical recovery” include outpatient treatment and more extended periods of lifetime mania (31). In contrast, higher baseline severity scores, presence of MDEs in the previous year, poor social functioning predict failure to achieve remission or recovery in BD (32). Among other clinical hallmarks, earlier (hypo-)manic or mixed recurrence, including sub-threshold manic symptoms and the proportion of days spent with an elevated mood in the preceding year, predict TRBD (33). The conceptualization of TRBD is more articulated than one of TRD, due to the differential natural and pharmacological course of BD and the controversial role of the antidepressants in BD (2). While drug resistance or worsening in MDD has been associated with mixed features and other bipolarity hints (34), relatively little is known about the impact of mixed features in bipolar depression and subsequence failure to respond. However, side-by-side comparisons of unipolar vs. bipolar MDEs relying on a relatively limited-range of retrospective data, suggesting that TDR represents a distinct psychopathological condition and not necessarily a prodromal state of BD (35).

The aim of the present exploratory-hypothesis generating study is to assess multiple clinical predictors of treatment resistance comparing unipolar vs. bipolar patients across different clinical subtypes of current MDE. To the best of our knowledge, the present data-driven study is the first of its kind to a side-by-side comparison of a range of putative clinical predictors of treatment resistance across different clinical subtypes of depression, either unipolar or bipolar major depression.

## Methods

### Participants

A hundred and thirty-one outpatients, both sexes, 18–65 years old, consecutively enrolled in a non-interventional study at a tertiary care unit of the Department of Psychiatry of the Federico II University of Naples, Italy, between May 3, 2017, and June 17, 2019. All patients met the Diagnostic and Statistical Manual for Mental Disorders, Fifth Edition (DSM-5) (36) criteria for a current MDE, either in the course of MDD or BD. Six experienced psychiatrists (MF, AF, SN, AA, ABl, PM) made the clinical diagnoses. Eligible patients signed a valid written informed consent after the study procedures had fully explained by the appointed principal investigator (MF). The protocol of the study was approved by the local I.R.B. on May 2^nd^, 2017, according to the 1964 Declaration of Helsinki and its subsequent amendments (37). Exclusion criteria were the lack of valid informed consent, untreated thyroid disease, schizophrenia, severe brain syndrome, or other organic conditions, potentially hampering the validity of the consent.

### Evaluation Procedure and Operational Definitions

The participating outpatients were rated according to the Hamilton Rating Scale for Depression, 17-item version scale (HAM-D-17) (38), Young Mania Rating Scale (YMRS) (39), and Temperament Evaluation of Memphis, Pisa, Paris, and San Diego, 110-item version (TEMPS-110) (40). The following clinical variables were recorded: age, sex, duration of the untreated illness (DUI), family history for header MDD or BD, lifetime suicidal behavior, significant psychiatric and medical comorbidities, and essential current and lifetime psychiatric treatment. The DUI was defined as the “time interval between the ﬁrst lifetime onset of symptoms of depression, not necessarily reaching the diagnostic threshed set by the DSM-5: at least five symptoms of depression, and the ﬁrst adequate treatment” (41). TRD was defined as “failure of two or more consecutive trials with antidepressants” (2). TRBD was defined as a lack of response to treatment to at least two previously established treatments for bipolar depression (e.g., olanzapine/fluoxetine combination, brexpiprazole, cariprazine, quetiapine, aripiprazole). TRBD could, however, have been exposed to at least one mood stabilizer drug plus an antidepressant in case of the previous failure to “adequate” trials for acute bipolar depression, especially whenever such therapy had been started elsewhere before seeking our consultation or in the presence of significant comorbidities (e.g., anxiety or obsessive–compulsive disorders).

Finally, the mixed features of the current MDE reflected the DSM-5 specifier. Besides, the “overlapping” symptoms otherwise excluded by the DSM-5 for a DSM-5-defined MDE with mixed features, i.e., “irritability,” “distractibility,” “impulsivity,” “mood lability,” and “psychomotor agitation,” were nonetheless recorded given their clinical relevance (42).

### Statistical Analysis

The statistical analysis was performed using IBM™ SPSS™ Statistics™ v.25.0.0 for Windows™ (43), adopting alpha.05, two-tailed. Non-parametric distributions were transformed as necessary. The interrater reliability for the analysis using Cohen's k statistics was ascertained for consistency issues. Descriptive statistics followed by principal component analysis (PCA) and by two-step cluster analysis were carried-out for MDD and BD patients, separately. Patients recorded as TRD or TRBD were considered as “cases”; those without a history of treatment resistance were deemed as “controls.” Oblique rotation solution (Promax) was performed by calculating the coefficient for each pair of variables, and multi-collinearity was excluded; coefficient ≤.3 were suppressed. We assumed the conservative loadings >.3 to be significant. The Kaiser–Meyer–Olkin measure of sampling adequacy resulted in.65, indicating an acceptable sample size, with Bartlett's test of sphericity scoring lower than.05. To determinate the component number, we considered only eigenvalues > 1. For readers' convenience, an eigenvalue of 1 would explain as much variance as the one explained by a single variable.

In plain, the PCA aimed to extract the psychopathological components captured by the HAM-D scale, while the cluster analysis carried aimed at discriminate the cases at study based on the PCA-fetched components. The generated clusters of depressions were compared against each other using descriptive statistics and binary logistic regression analysis.

## Results

### Demographic and Clinical Variables Across Different Clinical Subtypes of Depression

A summary of the essential clinical and demographic features of the included sample has been reported in **Table 1**. Essential current and lifetime pharmacological history are presented in **Table 2**, while **Table 3** provides the core rating tools and predominant affective temperament. Among other clinical variables, mixed, anxious, and psychotic features of current MDE were statistically more frequent among BD patients compared to the MDD counterpart.

**Table 1:** Essential clinical and demographics across unipolar and bipolar cases.

Study subjects (n = 131)	MDD (n = 78)	BD (n = 53, of whom BD-I = 24; BD-II = 29)	*t* or χ^2^ (df)	*p*
Essential demographics				
Age, (mean ± sd)	49.41 ± 13.90	50.30 ± 12.14	.379 (129)	.705
Sex F/M, n (%)	49 (37.4)/29 (22.1)	35 (26.7)/18 (13.7)	.142 (1)	.706
Ethnicity (Caucasian, other), n (%)	78 (59.5)	53 (40.5)	–	–
Primary school, n (%)	14 (10.7)	11 (8.4)	4.948 (3)	.176
Secondary school, n (%)	29 (22.1)	13 (9.9)		
High school, n (%)	29 (22.1)	19 (14.5)		
College, university, or higher education, n (%)	6 (4.6)	10 (7.6)		
Unmarried, n (%)	14 (10.7)	15 (11.5)	13.462 (2)	**.001**
Married/cohabiting, n (%)	61 (46.6)	27 (20.6)		
Widow/divorced, n (%)	3 (2.3)	11 (8.4)		
Essential lifetime comorbidities and clinical features				
Lifetime substance use disorder, n (%)	4 (3.1)	6 (4.6)	1.716 (1)	.190
Lifetime panic disorder, n (%)	28 (21.4)	17 (13)	.204 (1)	.651
Lifetime generalized anxiety disorder, n (%)	58 (44.3)	33 (25.2)	2.176 (1)	.140
Lifetime anorexia nervosa, n (%)	15 (11.5)	6 (4.6)	1.467 (1)	.226
Lifetime bulimia nervosa, n (%)	5 (3.8%)	4 (3.1)	.064 (1)	.801
Lifetime binge eating disorder, n (%)	8 (6.1%)	10 (7.6)	1.974 (1)	.160
Lifetime obsessive–compulsive disorder, n (%)	13 (9.9)	9 (6.9)	.002 (1)	.962
Lifetime specific phobia, n (%)	17 (13)	7 (5.3)	1.555 (1)	.212
Lifetime impulse control disorder, n (%)	5 (3.8)	20 (15.3)	20.054 (1)	**<.001**
Borderline personality disorder DSM-5, n (%)	3 (2.3)	5 (3.8)	1.718 (1)	0.190
History of previous psychiatric hospitalization, n (%)	25 (19.1)	29 (22.1)	6.691 (1)	**0.01**
Lifetime history suicidal behavior, n (%)	28 (21.4)	23 (17.6)	.746 (1)	.388
Duration of untreated illness, in years, (mean ± sd)	3.14 (3.77)	2.81 (5.61)	2.412 (129)	.81
Duration of current MDE, in weeks, (mean ± sd)	24.96 (40.34)	40.13 (84.06)	1.301 (114)	.196
Age at onset of depression (self-report), in years, (mean ± sd)	34.09 (14.32)	32.85 (14.49)	−.483 (129)	.630
Essential specifies				
DSM-5 MDE with melancholic features, n (%)	11 (8.4)	4 (3.1)	1.337 (1)	.247
DSM-5 MDE with mixed features, n (%)	3 (2.3)	7 (5.3)	3.922 (1)	**.048**
DSM-5 MDE with anxious distress, n (%)	33 (25.2)	13 (9.9)	4.378 (1)	**.036**
DSM-5 lifetime MDE with psychotic features, n (%)	3 (2.3)	9 (6.9)	6.543 (1)	**.001**
DSM-5 MDE with atypical features, n (%)	17 (13)	11 (8.4)	.020 (1)	.887
DSM-5 MDE with catatonic features, n (%)	0	0	–	–
Rapid cycling, course of depression, n (%)	1 (0.8)	5 (3.8)	4.798 (1)	.028
DSM-5 MDE with post-partum onset, n (%)	13 (14)	11 (11.8)	.331 (1)	.565

MDD, major depressive disorder; BD, bipolar disorder; DSM-5, Diagnostic and Statistical Manual of Mental Disorders, Fifth Edition; MDE, major depressive episode.

Note: Statistically significant difference in bold or p ≤.05.

**Table 2:** Essential pharmacological treatment history across the included cases.

		Current MDE			
		BD (any), n = 53		MDD, n = 78	
		TRBD or not		TRD or not	
		Not resistant = 38	TR(B)De = 15	Not resistant = 58	TRD = 20
Data expressed as “n (%)”					
Currently on antidepressant(s)		31 (81.59)	13 (86.67)	37 (63.79)	18 (90)
Currently on SGA(s)		21 (55.26)	6 (40)	1 (1.72)	12 (60)
Currently on lithium		13 (34.21)	11 (73.33)	4 (6.89)	10 (50)
Currently on FGA(s)		6 (15.79)	4 (26.67)	6 (10.34)	3 (15)
Currently on mood stabilizer(s) other than lithium		22 (57.89)	8 (53.33)	10 (17.24)	5 (25)
Currently on aripiprazole		3 (7.89)	4 (26.67)	0	2 (10)
Currently on asenapine		4 (10.53)	0	0	0
Currently on brexpiprazole		0	0	0	0
Currently on cariprazine		0	0	0	0
Currently on clozapine		0	0	0	0
Currently on lurasidone		0	0	0	0
Currently on olanzapine		4 (10.53)	2 (13.33)	0	2 (10)
Currently on paliperidone		0	0	0	0
Currently on quetiapine		9 (23.68)	0	1 (1.72)	7 (35)
Currently on risperidone		0	0	0	0
Currently on ziprasidone		1 (2.63)	0	0	0
Lifetime exposure to MOAI(s)		0	0	0	1 (5)
Lifetime exposure to carbamazepine		9 (23.68)	3 (20)	0	4 (20)
Lifetime exposure to SGA		26 (68.42)	12 (80)	7 (12.07))	16 (80)
Lifetime exposure to TCA		17 (44.74)	8 (53.33)	10 (17.24)	8 (40)
Lifetime exposure to valproate		25 (65.79)	10 (66.67)	12 (20.69)	9 (45)
Lifetime exposure to lithium		11 (28.95)	9 (60)	4 (6.89)	12 (60)
Lifetime psychotropic polypharmacy (four or more psychotropic drugs at once)		23 (60.53)	7 (46.67)	4 (6.89)	13 (65)

MDE, major depressive episode; MDD, major depressive disorder; BD, bipolar disorder; SGA, second-generation antipsychotic; FGA, first-generation antipsychotic; TCA, tricyclic antidepressant; MAOI, monoamine oxidase inhibitor; TRD, treatment-resistant depression; TRBD, treatment-resistant bipolar depression.

TRD = treatment resistant depression was defined as failure to respond to at least two adequate trials with antidepressant drugs. TRBD = treatment resistant bipolar depression was defined as lack of response to treatment to at least two previous established treatments for bipolar depression (e.g. olanzapine/fluoxetine combination, brexpiprazole, cariprazine, quetiapine, aripiprazole). Most of the bipolar depressed patients were currently in receipt of at least one mood stabilizer drug plus an antidepressant due to the high frequency of comorbid conditions, tough the antidepressant would not represent the established treatment for bipolar depression.

**Table 3:** Rating and temperamental profile of MDD and BD patients.

Study subjects (n = 131)	MDD (n = 78)	BD (n = 53, of whom BD-I = 24; BD-II = 29)	*t* or χ^2^ (df)	*p*
Essential ratings				
HAM-D-17 total score, (mean ± sd)	19.63 ± 6.19	19.55 ± 5.33	−.078 (129)	.334
YMRS item 2, “increased psychomotor activity” n (%)	3 (3.85)	42 (79.25)	10.935 (3)	**.012**
YMRS item 3, “hypersexuality” n (%)	1 (1.28)	7 (13.21)	7.827 (1)	**.005**
YMRS item 5, “irritable mood” n (%)	29 (31.18)	30 (56.6)	8.082 (3)	**.044**
YMRS item 7, “distractibility” n (%)	10 (12.82)	14 (26.42)	11.020 (2)	**.004**
TEMPS-A-110 domains for affective temperaments				
Depressive temperament domain score, range 1–21 (mean ± sd)	10.14 ± 4.04	10.58 ± 3.78	.568 (102)	.571
Cyclothymic temperament score, range 1–21 (mean ± sd)	6.64 ± 4.41	8.91 ± 4.44	2.590 (102)	**.011**
Hyperthymic temperament, score 1–21 (mean ± sd)	3.69 ± 3.21	5 ± 4.83	1.653 (102)	.101
Irritable temperament score, range 1–21 (mean ± sd)	4.08 ± 3.29	6.47 ± 4.37	3.170 (102)	**.002**
Anxious temperament score, range 1–21 (mean ± sd)	12.22 ± 6.2	12.22 ± 5.16	.002 (102)	.999

Statistically significant difference in bold or p ≤.05.

MDD, major depressive disorder; BD, bipolar disorder; HAM-D, Hamilton Depression Scale; YMRS, Young Mania Rating Scale; TEMPS, Temperament Evaluation of Memphis, Pisa, Paris, and San Diego-auto-questionnaire 110-item version.

Selected items of the YMRS emphasized herein as they represent the “overlapping” mixed features not accounted by the DSM-5 for the diagnosis of the “with mixed features” specifier of MDE.

### Principal Component and Cluster Analysis Statistics

PCA was performed on the items of the HAM-D-17 to characterize the sample at the report better, discriminating between unipolar and bipolar cases of depression. Two-step cluster analysis of cases based on the three components generated by the PCA led to two clinically interpretable clusters, both in the MDD (**Table 4**) and the BD (**Table 5**) group. Notably, the fetched clusters comprised different component-items from the original HAM-D scale across the BD and the MDD groups, as detailed in **Tables 4** and **5**, also reporting the proportion of resistant cases across different clusters, respectively.

**Table 4:** Clusters and their characterization generated by two-step cluster analysis based on the three components obtained upon principal component analysis (PCA) on MDD patients.

	Components generated by the PCA	Loading HAM-D-17 items	Interpretation of clusters	Patients, n (%)
Cluster 1	1	1, 2, 3, 4, 5, 6, 7, 8, 10, 15	Psychic symptoms, depressed mood, suicide, guilty, insomnia	22 (28.2), of whom 11 (14.1%) TRD
	2	12, 14, 16, 17	Genitourinary, gastrointestinal, weight loss, insight	
Cluster 2	3	9, 11, 13	Somatic anxiety and agitation	56 (71.8), of whom 9 (11.5%) TRD

HAM-D-17, Hamilton Depression Rating Scale, 17-item version.

**Table 5:** Clusters and their characterization generated by two-step cluster analysis based on the three components obtained upon Principal Component Analysis (PCA) on BD patient.

	Components generated by the PCA	Loading HAM-D-17 items	Interpretation of clusters	Patients, n (%)
Cluster 2	1	1, 2, 3, 5, 6, 7, 8, 10, 14	Psychic symptoms, depressed mood, suicide, guilty, insomnia, genitourinary symptoms	16 (30.2), of whom 5 (9.4%) TRBD
	2	11, 13, 15	Hypochondria, somatic anxiety and fatigue, headache	
Cluster 1	3	9, 12, 16, 17	Agitation, gastro-intestinal, weight loss, insight	37 (69.8), of whom 10 (18.9%) TRBD

HAM-D-17, Hamilton Depression Rating Scale, 17-item version.

### Prediction of Treatment Resistance Based on Clinical Subtyping of Depression and Mixed Features Across Unipolar and Bipolar Clusters

Overall, the clinical subtypes of depression fetched by cluster analysis failed to allow a conclusive distinction between resistant vs. non-resistant cases (please refer to **Tables 4** and **5** for details).

The presence of DSM-5-defined mixed features among MDD and BD cases was inversely related to the resistance outcome. In contrast, the presence of the “overlapping” symptoms of depression among MDD and BD—excluded by the DSM-5 specifiers of mixed depression—such as “distractibility,” “impulsivity,” “increased psychomotor activity,” and “hypersexuality,” predicted higher rates of treatment resistance, consistently in the MDD and the BD groups. Such “permissive” criteria for mixed depression, already outlined by the 1978 Research Diagnostic Criteria (44), consistently predicted treatment resistance both in the MDD and the BD groups even in the presence of different operational definitions and pharmacological courses differed between TRD and TRBD cases. Please refer to **Table 6** for details. Finally, in the MDD group, the DSM-5 mixed features and the broadly defined mixed features were endorsed by 1.5% and 6.1% of cluster 1 patients, and 0.8% and 6.1% of cluster 2 patients, respectively. In the BD group, group the DSM-5 mixed features and the broadly defined mixed features were disclosed by 7.6% and 12.2% of cluster 1 patients, and 0.8% and 1.5% of cluster two patients, respectively.

**Table 6:** Binary logistic regression, significant predictors only.

		B (S.E.)	Exp(B)	95% C.I. for EXP(B)	
				Lower bound	Upper bound
Major depression	Cluster 1 membership: Psychic symptoms, depressed mood, suicide, guilty, insomnia & genitourinary, gastro-intestinal, weight loss, insight	1.447 (.643)	**4.251**	1.206	14.983
	Mixed features (DSM-5 criteria)	−.281 (1.340)	.764*	.59	9.879
	Mixed features (permissive criteria)	2.409 (.683)	**11.124**	2.916	42.435
Bipolar depression	Mixed features (DSM-5 criteria)	−0.88 (9.29)	.916*	.148	5.658
	Mixed features (permissive criteria)	1.964 (.801)	**7.130**	1.485	34.247

In the MDD group, cases correctly classified = 80.8%, overall; Nagelkerke's R^2^ =.371. Cluster 2 membership among MDD cases (somatic anxiety and agitation) failed to reach the statistical significance threshold in the present model. In the BD group, cases correctly classified = 71.7%, overall; Nagelkerke's R^2^ =.194. “Permissive” refers to the standard DSM-5 specifier for mixed features of a major depressive episode broadened to include also the “overlapping” symptoms (e.g., irritability, impulsivity, distractibility) otherwise accounted by the Research Diagnostic Criteria (RDC).

*Note: For reader convenience, an odd ratio, OR—namely an Exp(B) in this instance—below one means that the given predictor is inversely related to the outcome at study (“protective” factor), whereas a value approximating 1 or equal to one means equal chances of the outcome at study occurring or not. All the bolded text indicate p <. 001.

## Discussion

Consistent with our hypothesis, the propensity toward TRD may vary across different clinical subtypes of depression. Cluster 1 membership among MDD cases, including the core depressive symptoms, psychomotor retardation, suicidal behavior (ideation or attempt), and somatic-anxiety (namely, gastrointestinal, indigestion, cardiovascular, palpitations, headaches, respiratory, and genitourinary symptoms), predicted TRD in half of such cases (14.1% of 28.2% of cluster 1 MDD patients). This is in line with previous evidence which, albeit failing to discriminate across different clinical subtypes of depression, highlighted the role of suicidal behavior—even those of mild to moderate intensity (27)—anxiety (25), and severity of the current MDE in the prediction of the treatment outcome (26, 28), as well as the impact of psychotic features of depression in the prediction of subsequent TRD (45). This is also in line with previous evidence involving patients diagnosed using the codes provided by the Fourth Edition of the Manual (DSM-IV) (46), yet failing to adopt a data-generated clustering of psychopathology (47). However, it must be noticed that the clustering had a poor predictive value overall in the MDD group and no significant predictive value for TRBD cases. Similarly, the affective temperaments measured by means of the TEMPS had no discriminative value for treatment resistance, neither in the MDD, neither in the BD subgroups, in line with previous reports comparing unipolar and bipolar cases (48).

On the other side, the present study highlights the impact of mixed features of depression, broadly defined to encompass also the not overlapping symptoms otherwise excluded by the DSM-5. Mixed features of depression were somewhat similar in terms of frequency among different data-generated subtypes of depression, in the MDD and the BD groups. Overall, mixed features consistently predicted treatment resistance among BD and MDD cases across different clinical subtypes of depression, especially when these latter included “excitement” symptoms among MDD patients. Indeed, mixed features of depression have been suggested to represent hallmarks of bipolarity among DSM-defined cases of BD, which may, on turn, be prone to treatment failure with standard MDD medications (49). In particular, MDD patients may require strategies other than the two “adequate” trials with antidepressants before receiving a diagnosis of treatment resistance in the presence of mixed features, especially when these latter encompass the “excitement” overlapping symptoms. In the MDD group, patients without broadly-defined mixed features, or DSM-5 defined mixed features of depression neither, may be more likely to respond to standard antidepressant care, thus endorsing lower rates of TRD. Stating the categorical nature of the operational definitions adopted by the present study for TRD or TRBD, it cannot be excluded, however, that the propensity for treatment resistance may span across a linear gradient ranging from almost neutral risk (OR =.916; C.I. =.148–5.658 in BD patients with narrowly-defined mixed features of depression) toward a 7-fold increased odd (OR = 7.130; C.I. = 1.485–34.247) in the BD group endorsing broadly-defined mixed features. Moreover, since the DSM-5-defined mixed features had negligible predictive value for treatment resistance, the use of the sole boundary-defined mixed features proposed by the DSM-5 itself may lead to improper pharmacological management of depression (50).

The categories proposed the DSM consist of “ideal types,” abstractions from reality, which we can use to assess empirical observations but which are not themselves, empirical facts. This latter concept, which is central to Jaspers' thinking in his textbook “General Psychopathology” (51), was derived from the work of Max Weber (52). While the ideal types may aid in discriminating depression from, say mania, the most common clinical presentations are an admixture of both conditions (53). From this perspective, we remark the relevance of the present study, although its preliminary nature solicits for additional research on the matter. However, more sensible operational definitions for TRD, especially in BD, are warranted (Fornaro M. et al., submitted for publication).

There several limitations of the present study, which was conducted in a tertiary care outpatient setting that needs to be accounted for in the interpretation of the results. The sample size is a relatively small one, with no longitudinal follow-up or systematic stratification of pharmacological history across different classes of antidepressants, titration modality. No stratification according to the predominant mood polarity or polarity index of BD was reported. Recall bias may have hampered the validity of some diagnoses, including general medical comorbidities (which may have nonetheless affected the propensity of treatment response). Specifically, those BD patients who were also in receipt of antidepressant(s) drugs beyond the mood stabilizer(s) need to be further stratified by upcoming studies in order to better account for several comorbidities (e.g., obsessive–compulsive disorder) that may have led to pseudo-resistance in some cases. However, per study-protocol, even those BD patients who were currently in receipt of antidepressant(s) at the time of the assessment had already failed at least two adequate courses with established treatments for acute bipolar depression (e.g., the atypical antipsychotic quetiapine, or the olanzapine/fluoxetine combination). Besides, there was no virtually no record of the psychosocial or neuro-modulatory interventions, any correlation measures with neither neuroimaging, molecular nor genetic marker. Yet, neuro-modulatory interventions such as electroconvulsive therapy represent consolidated treatment avenues for TRD (54). Besides, the psychopathological clustering of depression based entirely on the sole HAM-D, which may not be sufficient to catch the complexity of depression exhaustively.

However, stating the data-generating hypothesis of the present preliminary report, we further reinforce the need for upcoming studies to replicate the present results across different clinical subtypes of depression and a large number of putative biological and neuropsychological measures.

## Data Availability Statement

The raw data supporting the conclusions of this article will be made available by the authors, without undue reservation, to any qualified researcher.

## Ethics Statement

The studies involving human participants were reviewed and approved by Federico II University of Naples. The patients/participants provided their written informed consent to participate in this study.

## Author Contributions

MF and AF conceived the study and extensively edited the main-text and its attachments together with SN, ABl, AA, PM, FI, and ABa. Finally, all the co-authors contributed substantially to the present piece of work.

## Conflict of Interest

The authors declare that the research was conducted in the absence of any commercial or financial relationships that could be construed as a potential conflict of interest.

## References

[1] Malhi G Parker G Crawford J Wilhelm K Mitchell P Treatment-resistant depression: resistant to definition? Acta Psychiatr Scand (2005) 112(4):302–9. 10.1111/j.1600-0447.2005.00602.x 16156838

[2] McIntyre RS Filteau M-J Martin L Patry S Carvalho A Cha DS Treatment-resistant depression: definitions, review of the evidence, and algorithmic approach. J Affect Disord (2014) 156:1–7. 10.1016/j.jad.2013.10.043 24314926

[3] Hui Poon S Sim K Baldessarini RJ Pharmacological approaches for treatment-resistant bipolar disorder. Curr Neuropharmacol (2015) 13: (5):592–604. 10.2174/1570159X13666150630171954 26467409 PMC4761631

[4] Mrazek DA Hornberger JC Altar CA Degtiar I A review of the clinical, economic, and societal burden of treatment-resistant depression: 1996–2013. Psychiatr Serv (2014) 65(8):977–87. 10.1176/appi.ps.201300059 24789696

[5] Sylvia LG Shelton RC Kemp DE Bernstein EE Friedman ES Brody BD Medical burden in bipolar disorder: findings from the Clinical and Health Outcomes Initiative in Comparative Effectiveness for Bipolar Disorder study (Bipolar CHOICE). Bipolar Disord (2015) 17(2):212–23. 10.1111/bdi.12243 25130321

[6] Greenberg PE Birnbaum HG The economic burden of depression in the US: societal and patient perspectives. Expert Opin Pharmacother (2005) 6(3):369–76. 10.1517/14656566.6.3.369 15794728

[7] Johnston KM Powell LC Anderson IM Szabo S Cline S The burden of treatment-resistant depression: A systematic review of the economic and quality of life literature. J Affect Disord (2019) 242:195–210. 10.1016/j.jad.2018.06.045 30195173

[8] Kemp AH Gordon E Rush AJ Williams LM Improving the prediction of treatment response in depression: integration of clinical, cognitive, psychophysiological, neuroimaging, and genetic measures. CNS Spectrums (2008) 13: (12):1066–86. 10.1017/S1092852900017120 19179943

[9] Vergunst FK Fekadu A Wooderson SC Tunnard CS Rane LJ Markopoulou K Longitudinal course of symptom severity and fluctuation in patients with treatment-resistant unipolar and bipolar depression. Psychiatry Res (2013) 207(3):143–9. 10.1016/j.psychres.2013.03.022 23601791

[10] Kim Y-K Major Depressive Disorder: Current Advances and Paradigm Shifts. Psychiatry Invest (2020) 17(3):179–80. 10.30773/pi.2019.0092 PMC7113174 32209964

[11] Sanches M Quevedo J Soares JC Treatment Resistance in Bipolar Disorders. In: Treatment Resistance in Psychiatry. New York, USA (2019). p. 139–50.

[12] Bennabi D Aouizerate B El-Hage W Doumy O Moliere F Courtet Risk factors for treatment resistance in unipolar depression: a systematic review. J Affect Disord (2015) 171:137–41. 10.1016/j.jad.2014.09.020 25305428

[13] Coplan JD Gopinath S Abdallah CG Berry BR A neurobiological hypothesis of treatment-resistant depression–mechanisms for selective serotonin reuptake inhibitor non-efficacy. Front Behav Neurosci (2014) 8:189. 10.3389/fnbeh.2014.00189 24904340 PMC4033019

[14] Caraci F Calabrese F Molteni R Bartova L Dold M Leggio GM International union of basic and clinical pharmacology CIV: The neurobiology of treatment-resistant depression: From antidepressant classifications to novel pharmacological targets. Pharmacol Rev (2018) 70(3):475–504. 10.1124/pr.117.014977 29884653

[15] Muneer A The neurobiology of bipolar disorder: an integrated approach. Chonnam Med J (2016) 52(1):18–37. 10.4068/cmj.2016.52.1.18 26865997 PMC4742607

[16] Andreazza AC Young LT The neurobiology of bipolar disorder: identifying targets for specific agents and synergies for combination treatment. Int J Neuropsychopharmacol (2014) 17(7):1039–52. 10.1017/S1461145713000096 23449044

[17] Harrison PJ Geddes JR Tunbridge EM The emerging neurobiology of bipolar disorder. Trends Neurosci (2018) 41(1):18–30. 10.1016/j.tins.2017.10.006 29169634 PMC5755726

[18] Mrazek DA Lerman CJJ Facilitating clinical implementation of pharmacogenomics. JAMA (2011) 306: (3):304–5. 10.1001/jama.2011.1010 PMC3189505 21771991

[19] Perlis RH A clinical risk stratification tool for predicting treatment resistance in major depressive disorder. Biol Psychiatry (2013) 74(1):7–14. 10.1016/j.biopsych.2012.12.007 23380715 PMC3690142

[20] Hidalgo-Mazzei D Berk M Cipriani A Cleare AJ Di Florio A Dietch D Treatment-resistant and multi-therapy-resistant criteria for bipolar depression: consensus definition. Br J Psychiatry (2019) 214(1):27–35. 10.1192/bjp.2018.257 30520709 PMC7613090

[21] Schosser A Serretti A Souery D Mendlewicz J Zohar J Montgomery S European Group for the Study of Resistant Depression (GSRD)—where have we gone so far: review of clinical and genetic findings. Eur Neuropsychopharmacol (2012) 22(7):453–68. 10.1016/j.euroneuro.2012.02.006 22464339

[22] Bartova L Dold M Kautzky A Fabbri C Spies M Serretti A Results of the European Group for the study of resistant depression (GSRD)—basis for further research and clinical practice. World J Biol Psychiatry (2019) 20(6):427–48. 10.1080/15622975.2019.1635270 31340696

[23] Souery D Oswald Massat I Bailer U Bollen J Demyttenaere K Clinical factors associated with treatment resistance in major depressive disorder: results from a European multicenter study. J Clin Psychiatry (2007) 68(7):1062–70. 10.4088/JCP.v68n0713 17685743

[24] Gratten J Wray NR Keller MC Visscher PM Large-scale genomics unveils the genetic architecture of psychiatric disorders. Nat Neurosci (2014) 17(6):782. 10.1038/nn.3708 24866044 PMC4112149

[25] Dold M Bartova L Souery D Mendlewicz J Serretti A Porcelli S Clinical characteristics and treatment outcomes of patients with major depressive disorder and comorbid anxiety disorders-results from a European multicenter study. J Psychiatr Res (2017) 91:1–13. 10.1016/j.jpsychires.2017.02.020 28284107

[26] Kautzky A Baldinger-Melich Kranz GS Vanicek T Souery D Montgomery S A New Prediction Model for Evaluating Treatment-Resistant Depression. J Clin Psychiatry (2017) 78(2):215–22. 10.4088/JCP.15m10381 28068461

[27] Dold M Bartova L Fugger G Kautzky A Souery D Mendlewicz J Major depression and the degree of suicidality: results of the European Group for the Study of Resistant Depression (GSRD). Int J Neuropsychopharmacol (2018) 21(6):539–49. 10.1093/ijnp/pyy009 PMC6007240 29860382

[28] Kautzky A Dold M Bartova L Spies M Kranz GS Souery D Clinical factors predicting treatment resistant depression: affirmative results from the European multicenter study. Acta Psychiatr Scand (2019) 139(1):78–88. 10.1111/acps.12959 30291625 PMC6586002

[29] Pigoni A Delvecchio G Madonna D Bressi C Soares J Brambilla Can Machine Learning help us in dealing with Treatment Resistant Depression? A review. J Affect Disord (2019) 259:21–6. 10.1016/j.jad.2019.08.009 31437696

[30] Kautzky A Dold M Bartova L Spies M Vanicek T Souery D Refining Prediction in Treatment-Resistant Depression: Results of Machine Learning Analyses in the TRD III Sample. J Clin Psychiatry (2018) 79(1). 10.4088/JCP.16m11385 29228516

[31] Dikeos D Badr MG Yang F Pesek MB Fábián Z Tapia-Paniagua G Twelve-month prospective, multinational, observational study of factors associated with recovery from mania in bipolar disorder in patients treated with atypical antipsychotics. World J Biol Psychiatry (2010) 11(4):667–76. 10.3109/15622970903544638 20334575

[32] Haro J Reed C Gonzalez-Pinto A Novick D Bertsch J Vieta E 2-Year course of bipolar disorder type I patients in outpatient care: factors associated with remission and functional recovery. Eur Neuropsychopharmacol (2011) 21(4):287–93. 10.1016/j.euroneuro.2010.08.001 20956071

[33] Perlis RH Ostacher MJ Patel JK Marangell LB Zhang H Wisniewski SR Predictors of recurrence in bipolar disorder: primary outcomes from the Systematic Treatment Enhancement Program for Bipolar Disorder (STEP-BD). Am J Psychiatry (2006) 163(2):217–24. 10.1176/appi.ajp.163.2.217 16449474

[34] Perugi G Pacchiarotti I Mainardi C Verdolini N Menculini G Barbuti M Patterns of response to antidepressants in major depressive disorder: Drug resistance or worsening of depression are associated with a bipolar diathesis. Eur Neuropsychopharmacol (2019) 29(7):825–34. 10.1016/j.euroneuro.2019.06.001 31227264

[35] Nuñez NA Comai S Dumitrescu E Ghabrash MF Tabaka J Saint-Laurent M Psychopathological and sociodemographic features in treatment-resistant unipolar depression versus bipolar depression: a comparative study. BMC Psychiatry (2018) 18(1):68. 10.1186/s12888-018-1641-y 29548306 PMC5857132

[36] APA Diagnostic and statistical manual of mental disorders (DSM-5®). Association AP, editor. Washington D.C: American Psychiatric Publishing (2013).

[37] WMA World Medical Association Declaration of Helsinki: ethical principles for medical research involving human subjects. JAMA (2013) 310(20):2191–4. 10.1001/jama.2013.281053 24141714

[38] Hamilton M A rating scale for depression. J Neurol Neurosurg Psychiatry (1960) 23:56–62. 10.1136/jnnp.23.1.56 14399272 PMC495331

[39] Young RC Biggs JT Ziegler VE Meyer DA A rating scale for mania: reliability, validity and sensitivity. Br J Psychiatry (1978) 133(5):429–35. 10.1192/bjp.133.5.429 728692

[40] Akiskal HS Akiskal KK Haykal RF Manning JS Connor PD TEMPS-A: progress towards validation of a self-rated clinical version of the Temperament Evaluation of the Memphis, Pisa, Paris, and San Diego Autoquestionnaire. J Affect Disord (2005) 85: (1-2):3–16. 10.1016/j.jad.2004.12.001 15780671

[41] Dell'Osso B Altamura AC Duration of untreated psychosis and duration of untreated illness: new vistas. CNS Spectr (2010) 15(4):238–46. 10.1017/S1092852900000079 20414173

[42] Perugi G Angst J Azorin J-M Bowden CL Mosolov S Reis J Mixed features in patients with a major depressive episode: the BRIDGE-II-MIX study. J Clin Psychiat (2015) 76: (3):e351–8. 10.4088/JCP.14m09092 25830457

[43] IBM IBM SPSS Statistics for Windows, Version 25.0. Armonk, NY: IBM (2017).

[44] Spitzer RL Endicott J Robins E Research diagnostic criteria: rationale and reliability. Arch Gen Psychiatry (1978) 35: (6):773–82. 10.1001/archpsyc.1978.01770300115013 655775

[45] Dold M Bartova L Kautzky A Porcelli S Montgomery S Zohar J Psychotic Features in Patients With Major Depressive Disorder: A Report From the European Group for the Study of Resistant Depression. J Clin Psychiatry (2019) 80(1). 10.4088/JCP.17m12090 30677267

[46] Diagnostic and Statistical Manual of Mental Disorders, Fourth Edition (DSM-IV). Washington D.C: A.P. Association (2010).

[47] Arnow BA Blasey C Williams LM Palmer DM Rekshan W Schatzberg AF Depression subtypes in predicting antidepressant response: a report from the iSPOT-D trial. Am J Psychiatry (2015) 172: (8):743–50. 10.1176/appi.ajp.2015.14020181 25815419

[48] Zaninotto L Souery D Calati R Di Nicola M Montgomery S Kasper S Temperament and character profiles in bipolar I, bipolar II and major depressive disorder: Impact over illness course, comorbidity pattern and psychopathological features of depression. J Affect Disord (2015) 184:51–9. 10.1016/j.jad.2015.05.036 26070046

[49] Zaninotto L Souery D Calati R Scudellari Janiri L Montgomery S Mixed, melancholic, and anxious features in depression: a cross-sectional study of sociodemographic and clinical correlates. Ann Clin Psychiatry (2014) 26(4):243–53. 25166487

[50] Malhi GS Das Mannie Z Irwin L Treatment-resistant depression: problematic illness or a problem in our approach? Br J Psychiatry (2019) 214(1):1–3. 10.1192/bjp.2018.246 30565539

[51] Jaspers K General psychopathology Vol. 2 Baltimore, Maryland, USA (1997).

[52] Gerth HH Mills CW From Max Weber: essays in sociology. Boston, MA, USA (2014).

[53] Ghaemi SN Feeling and time: the phenomenology of mood disorders, depressive realism, and existential psychotherapy. Schizophr Bull (2006) 33(1):122–30. 10.1093/schbul/sbl061 PMC2632297 17122410

[54] Kraus C Kadriu B Lanzenberger R Zarate CA Jr. Kasper S Prognosis and improved outcomes in major depression: a review. Trans Psychiatry (2019) 9(1):1–17. 10.1038/s41398-019-0460-3 PMC6447556 30944309

